# Assessing unprofessional behaviors of dental students through multi-source feedback: A mixed methods analysis of engagement and influencing factors

**DOI:** 10.1371/journal.pone.0353159

**Published:** 2026-07-17

**Authors:** Maryam Kazemipoor, Shima Habibzadeh, Fatemeh Keshmiri

**Affiliations:** 1 Department of Endodontics, School of Dentistry, Shahid Sadoughi University of Medical Sciences, Yazd, Iran; 2 Student Research Committee, Shahid Sadoughi University of Medical Sciences, Yazd, Iran; 3 Medical Education Department, Education Development Center, Shahid Sadoughi University of Medical Sciences, Yazd, Iran; 4 Research Development Center, Shahid Sadoughi Hospital, School of Medicine, Shahid Sadoughi University of Medical Sciences, Yazd, Iran; Universidade dos Açores Departamento de Biologia: Universidade dos Acores Departamento de Biologia, PORTUGAL

## Abstract

**Background:**

Unprofessional behaviors have been identified as a hindrance to the professional identity formation of dental students. This study aimed to investigate dental students’ engagement with unprofessional behavior in a clinical setting and to explore their experiences regarding the factors influencing this behavior in dental school.

**Methods:**

The sequential mixed-methods study conducted at Shahid Sadoughi University of Medical Sciences. A total of 140 dental students participated, selected through a census method. The quantitative phase focused on evaluating unprofessional behaviors among students using Multi-Source Feedback (MSF), which included assessments from clinical teachers, nurses, and self-evaluations by the students. In the qualitative phase, participants were chosen through purposeful sampling, and their experiences were explored through semi-structured interviews.

**Results:**

Responses from all three groups of clinical teachers, nurses, and students showed that the highest students’ engagement with unprofessional behaviors was related to aspects of responsibility and respect. The highest engagement of unprofessional behavior was reported among junior learners (P = 0.0001) and male students (P = 0.0001). The students’ experiences were classified into a theme “disturbance of individual and systemic factors in the development of professional behaviors,” with two categories of “individualism” and “challenges of the overt and covert curriculum in professional development”.

**Conclusion:**

The disruption of both individual and systemic factors has led to unprofessional behaviors at the dental school. On an individual level, issues such as a focus on profit and a reluctance to meet professional responsibilities have hindered adherence to principles of professionalism. At the systemic level, negative role models and inadequate educational requirements and training processes have also contributed to the prevalence of unprofessional behaviors among participants. Given generalizability limited to culturally similar educational contexts, other institutions may adopt MSF and targeted interventions on clinical role models and early responsibility training.

## 1. Introduction

Professionalism is a crucial capability in medical education, encompassing the norms, behaviors, and values that build trust in health providers [1,2]. Adherence to professionalism principles is crucial in forming personal and professional identity [3,4]. The General Dentistry Association of London defines dental professional standards in various areas, including giving priority to the patient’s interests and trying to protect them, respecting the patients and their choices, maintaining the confidentiality of the patient’s information, developing professional capabilities, and the ability to build trust and cooperation with the dental team members to provide services to the patient [5].

The American Dental Education Association identifies six key elements of professionalism in dentistry: competence, honesty, responsibility, justice, respect, and service-mindedness [1]. The General Dental Council (GDC) outlines nine principles of professionalism that include both a personal and an organizational dimension. The personal dimension emphasizes prioritizing patient interests, fostering effective communication, and maintaining patient trust through appropriate behavior. The organizational dimension highlights the importance of confidentiality regarding patient information [5]. Further, Nguyen and colleagues define professionalism as a commitment to ethical principles, which encompass respect for patient autonomy, beneficence, and social justice [6].

Professionalism, as a meta-skill and second-order competence, manifests in various dimensions of communication with patients, colleagues, and the community in the dental profession [1]. Unprofessional behaviors in recent years have hindered the development of professionalism among members of different health professions and the formation of professional identity among students in educational systems [7]. Unprofessional behavior refers to a professional’s irregular actions when providing services in educational and treatment settings. In a systematic review, Mak-Van categorized the unprofessional behaviors of medical students into four main themes: failure to engage, dishonest behavior, disrespectful behavior, and a lack of self-awareness [7]. These behaviors disrupt relationships among patients, healthcare team members, and leaders, such as doctors and dentists. Van Mook and colleagues reported that unprofessional behaviors negatively impacted treatment outcomes [8].

Papadakis et al demonstrated a significant positive association between undergraduate students’ engagement in unprofessional behaviors during their academic training and the persistence of such behaviors in their subsequent professional careers [9]. This situation highlights the critical need to accurately identify the specific components of unprofessional conduct and clarify the underlying factors that lead students to exhibit such behaviors in educational settings [10–13]. Despite growing recognition of this issue, there remains a substantial knowledge gap concerning the contextual and systemic determinants that facilitate the emergence and continuation of unprofessional behaviors in academic settings, thereby highlighting an urgent need for further empirical investigation and targeted interventions [7,12,14]. It is recommended to conduct further studies to understand unprofessional behaviors and the influencing factors [2,15–17].

Although numerous studies have examined unprofessional behavior in medicine [12,13,18–20], there remains a need for further research specifically addressing unprofessional conduct within the field of dentistry [11]. The present study addresses this disparity by employing multi-source feedback (MSF) to systematically evaluate unprofessional behaviors among dental students, thereby contributing to a more nuanced understanding within this specialized field.

This study aimed to investigate dental students’ engagement with unprofessional behavior in a clinical setting using the Multi-Source Feedback (MSF) method. It also explored the students’ experiences regarding the factors that influence their involvement with unprofessional behavior in dental school.

## 2. Method

This is a sequential mixed-method study involving two phases, quantitative and qualitative, conducted at Shahid Sadoughi University of Medical Sciences between the10 February 2021 and 23 February 2022.

### 2.1. Study participants/sample

#### 2.1.1. Student.

The inclusion criteria were the students who had experienced working in clinical departments and studying from the 7^th^ to the 12^th^ semester in the dental school. In the quantitative phase, the students were included in the census (n = 140), and in the qualitative phase, students were participants through a purposeful sampling (n = 15).

The inclusion criteria for the quantitative phase of the study included 140 dental students in Semesters 7–12, selected by census, and who had worked in clinical departments at the dental school of the university.

A purposive sampling strategy was used to recruit 15 students from the initial 140 to participate in the second, qualitative phase of the research. Participants were eligible for this phase if they had reported a high frequency of unprofessional behavior in the first phase.

#### 2.1.2. Evaluators.

A total of 48 evaluators participated in this study. To ensure diversity across the educational environment, evaluators were recruited from 12 academic departments within the dental school. The criteria for participation in the study included: (a) completion of a training workshop on implementing the evaluation program, (b) at least four years of teaching experience, (c) observation of students in a minimum of six educational sessions, and (d) willingness to participate in the study. From each department, four evaluators who met all the criteria were selected: two faculty members (one male and one female) and two nurses (one male and one female). All participants were chosen based on their availability and willingness to collaborate. The final sample comprised 48 evaluators, including 24 faculty members and 24 nurses (see Table 1).

**Table 1:** The demographic characteristics of students and evaluators.

Students			Evaluators	
Quantitative phase		Qualitative phase	Quantitative phase	
**Gender** N (%)				
Men	28 (20)	7 (46.6)	24 (50)	
Women	112 (80)	8 (53.3)	24 (50)	
**Age** Mean (±SD)	23.77 (±1.93)	24 ± 3	46 ± 5	
**Academic Semesters** N (%)			**Position** N (%)	
7^th^ semester	27 (19.3)	2 (13.33)	Faculty members	24 (50)
8^th^ semester	15 (10.7)	3 (20)	Nurses	24 (50)
9^th^ semester	30 (21.4)	3 (20)		
10^th^ semester	23 (16.4)	2 (13.33)		
11^th^ semester	21 (15)	2 (13.33)		
12^th^ semester	24 (17.1)	3 (20)		

#### 2.1.3. Interviewer.

The interviewer held a dental degree and had completed extensive training in qualitative interviewing techniques before collecting data. To maintain methodological rigor, the qualitative interviews were conducted by a trained interviewer who was independent of the evaluators involved in the quantitative phase. This independence, along with the specialized training, improved the credibility and trustworthiness of the data.

### 2.2. Data collection implementation

#### 2.2.1. Study instrument.

i) **Quantitative Phase**

The MSF instrument consisted of a 28-item questionnaire used to assess students’ unprofessional behaviors. Each item was scored in a binary manner, where “no” was assigned a score of 0 and “yes” a score of 1. The questionnaire included demographic questions about the students, such as age, gender, and academic semester, as well as five binary questions regarding their previous participation in professional courses and their familiarity with the concepts of medical ethics and professionalism. This questionnaire was originally developed in Persian by Jamal Abadi et al. for use in medical education, achieving a Cronbach’s α of 0.79 [21]. Since the current study was conducted in the context of dental education, the instrument’s psychometric properties were re-evaluated to confirm its suitability for this specific population. This evaluation took place before the main data collection and included three stages: qualitative content validity, quantitative content validity, and reliability assessment.

To evaluate the qualitative content validity of the questionnaire in the field of dentistry, we consulted a panel of 16 independent experts, all of whom are dental clinical teachers. Each expert was asked to review the questionnaire individually and provide feedback on the relevance, clarity, and comprehensiveness of the items about dental education. Their comments were collected and summarized for discussion at a subsequent meeting of the expert panel. As a result of their feedback, one item was removed from the original 28-item questionnaire.

Following the qualitative review, the quantitative indicators of content validity—specifically the Content Validity Ratio (CVR) and the Content Validity Index (CVI)—were calculated [22,23]. The CVR was assessed by asking the same panel of 16 experts to rate each item individually on a three-point scale: (1) essential, (2) useful but not essential, and (3) not essential. The minimum acceptable value for the CVR was determined based on Lawshe’s table [24]. The CVI was used to assess the relevance of each item to the construct of professionalism in dentistry [24]. The experts individually rated the relevance of each item using a four-point Likert scale. For this study, we calculated the item-level content validity index (I-CVI) for each item, as well as the scale-level content validity index (S-CVI/Ave), which is the average of the I-CVIs for all items. When there are more than five experts, a minimum I-CVI of 0.79 is regarded as acceptable according to standard recommendations [23]. The results of both the CVR and CVI analyses, along with the qualitative feedback, were reviewed and discussed in a joint session with the expert panel to finalize the questionnaire items.

The reliability of the revised questionnaire was assessed by examining its internal consistency. For this pre-testing phase, a separate sample of 35 dental students was recruited. These 35 students were distinct from and not included in the main study sample of 140 participants. This sample consisted of 15 men (42.85%) and 20 women (57.14%), with a mean age of 27 years (SD = 3). The questionnaire was administered to this group, and the Cronbach’s alpha coefficient was calculated to confirm its reliability for the dental student population.

ii) **Qualitative phase:**

The data collection for the qualitative phase of the study was a semi-structured interview guide. This guide was developed by the research team based on the study’s objectives and a review of existing literature on professionalism and unprofessional behavior in medical and dental education. An initial draft of the interview guide was reviewed and refined by a panel of five experts in dental education and qualitative research to ensure its relevance and comprehensiveness. The final version of the interview guide aimed to explore participants’ experiences within a main domain, which was derived from both the quantitative phase findings and an extensive review of the literature on professionalism in medical and dental education. This main domain focused on the factors that influence engagement in unprofessional behavior. It sought to investigate the underlying reasons contributing to unprofessional behaviors, particularly emphasizing individual factors (such as personal attitudes, stress, burnout, and knowledge gaps) as well as contextual factors (including the clinical environment, curriculum, role modeling, peer influence, and institutional culture).

The interview began with the primary open-ended question: *“Have you ever observed unprofessional behavior in dental school? What factors do you believe lead you or your classmates to engage in unprofessional behavior?”* Depending on the responses, additional probing questions, such as *“Can you tell me more about that?”* or *“What do you mean by that?”* were asked to facilitate deeper discussion. The interview guide is provided in Appendix 1.

#### 2.2.2. Data Collection.

In this mixed-methods study, data were collected sequentially in two distinct phases: a quantitative phase based on Multi-Source Feedback (MSF), followed by a qualitative phase using semi-structured interviews. Each phase was implemented according to a predefined protocol to ensure methodological rigor and transparency.

i) **Quantitative Phase**

The quantitative component utilized a 360-degree Multi-Source Feedback (MSF) approach to evaluate students’ professional behaviors in clinical settings. MSF is a structured performance assessment method where various stakeholder groups assess predefined behaviors using standardized rating instruments.

**Evaluators and Sources of Assessment**: Three groups participated in the MSF process: 1) clinical faculty members, 2) clinical nurses, and 3) dental students (for self-assessment).

**Evaluator Training:** To enhance inter-rater reliability and minimize systematic bias, all evaluators underwent a structured training workshop before data collection. This training included: clarification of the objectives and conceptual framework of MSF, a detailed review of competency domains and behavioral descriptors, calibration exercises using standardized clinical vignettes, and guidance on providing performance-based, constructive feedback. This structured preparation aimed to improve rating consistency and promote a shared understanding of performance standards.

**Evaluation Procedure for Clinical Teachers and Nurses:** Faculty members and nurses evaluated students based on direct observations of their professional conduct in real clinical contexts. They used the 28-item questionnaire as an observation-based assessment tool to rate the frequency of unprofessional behaviors they observed in students during clinical rotations. To ensure adequate exposure, evaluators were instructed to complete the assessment forms only after observing each student for a minimum of two consecutive weeks. The evaluation forms were then distributed to eligible evaluators, who completed and returned them directly to the research team to maintain confidentiality and reduce social desirability bias. These ratings were then analyzed alongside students’ self-assessments to provide a multi-source perspective on unprofessional behaviors.

**Self-Assessment Procedure:** Students completed the same instrument as a self-report measure, allowing for comparison between their self-perception and the external evaluations.

ii) **Qualitative phase:**

After the quantitative phase, semi-structured interviews were conducted to explore students’ experiences and perceptions regarding factors that contribute to unprofessional behavior.

**Interviewer:** A female interviewer, who holds a dental degree and is independent of the evaluators from the quantitative phase, conducted all interviews. She received thorough training in qualitative interviewing techniques before data collection.

**Interview Setting:** The timing and location of each interview were arranged to fit the students’ schedules. All interviews took place in a quiet, private room at the School of Dentistry to ensure confidentiality and minimize distractions.

Before starting each interview, the interviewer explained the purpose of the research, the interview method, and the voluntary nature of participation. Participants were assured that their information would be kept confidential and informed that written consent had been obtained from each individual.

**Interview Process:** Each interview began with the main question from the semi-structured interview guide: *“Have you ever observed unprofessional behavior in dental school? What factors do you believe lead you or your classmates to behave unprofessionally?”* Based on the participants’ responses, the interviewer used probing questions (e.g., *“Can you tell me more about that?”*, *“What do you mean by that?”*, *“Can you give a specific example?”*) to encourage deeper reflection and to clarify their experiences.

**Data Collection Duration:** The interviews lasted, on average, approximately 45 minutes. All interviews were audio-recorded with the participants’ permission. During the interviews, the interviewer also took notes on initial impressions and non-verbal cues.

**Data Saturation:** Data collection continued until no new codes or themes emerged from the interviews, indicating that data saturation had been reached.

#### 2.2.3. Data management and analysis.

**Quantitative Analysis**: **Data Management:** Quantitative data were collected using paper-based questionnaires completed by clinical teachers, nurses, and students. Each questionnaire was assigned a unique code to ensure participant confidentiality. Data were manually entered into IBM SPSS Statistics software (version 22) by a researcher independently to verify accuracy and minimize entry errors. A second researcher cross-checked a random sample of 20% of the entered data against the original questionnaires to ensure data integrity.

**Data Analysis:** Descriptive statistics, including frequencies, percentages, means, and standard deviations, were calculated to summarize participants’ demographic characteristics and the prevalence of unprofessional behaviors across the three rater groups (clinical teachers, nurses, and students). In addition, data were analyzed by the Mann-Whitney test, Friedman test, and Bonferroni adjustment as a post-hoc analysis, and Pearson’s correlation coefficient. Data were analyzed with SPSS 22. The significance level was P-value < .05

**Qualitative Analysis**: **Data Management:** All audio-recorded interviews were transcribed verbatim in Persian within 48 hours of each interview to ensure accuracy and preserve contextual nuances. Each transcript was assigned a code to protect participant anonymity. To enhance data trustworthiness, transcripts were returned to a subset of participants for member checking, allowing them to confirm or clarify their responses.

**Data Analysis:** To achieve immersion in the data, the researchers listened to the interviews several times and reviewed their transcriptions. The conventional method of Graneheim and Lundman was used to analyze the data. Based on this approach, data coding starts with open codes, and categories and more abstract themes emerge by combining categories [25]. Semantic units that expressed the students’ experiences were extracted from the participants’ statements. The open codes were subsequently merged and placed in categories based on semantic affinity. After organizing them based on their relationship, the categories were formed. The theme emerged by comparing the similarities and differences of the categories.

**Trustworthiness**: Schwandt’s criteria were adhered to ensure the rigor of data in this study [26]. Several methods were employed to ensure the credibility of the data. This included reflecting on the research’s purpose and main question, as well as conducting in-depth interviews with open-ended questions to gather information. We also engaged in a thorough analysis of the semantic units, maintained long-term involvement with the data, and dedicated sufficient time to both data collection and analysis. The interviews and their corresponding results were reviewed and approved by the participants, ensuring member checking. Furthermore, the data analysis process and the exploration of findings were carefully examined by experienced members of the research team specializing in qualitative research. Moreover, two experts in qualitative studies audited and confirmed the findings (expert checking). To maintain coherence, continuous comparisons were made between categories and subcategories regarding their semantic and structural integrity. To enhance the transferability of the findings, we provided a detailed description of the context, participant selection process, participant characteristics, data collection methods, and data analysis procedures.

### 2.3. Ethical Considerations

This study was approved by the Committee of Ethics at Shahid Sadoughi University of Medical Sciences. Yazd, Iran (ID: IR.SSU.REC.1398.225). Informed written consent was obtained from the participants. Individuals’ characteristic information and recorded data were kept confidential. The study’s objectives were explained to the participants, who were allowed to enter and refuse the study voluntarily at any time.

## 3. Results

### 3.1. Results of Quantitative phase

#### 3.1.1. Validation of the instrument.

The CVR value for all items was higher than the minimum acceptable value, so all items were kept in the questionnaire. According to the I-CVI index, the CVI values of all competencies were above 0.79 and were maintained in the framework. Quantitative validity results for the questionnaire were confirmed by S-CVI/ Ave = 0.83 (scale-level content validity index). The reliability of the questionnaire in dentistry was confirmed with a Cronbach’s alpha of 0.89. The validation of the 28-item questionnaire has been approved. (Appendix 2).

#### 3.1.2. MSF evaluation.

Table 1 shows the demographic characteristics of students and evaluators.

The frequency of unprofessional behaviors of learners in responses to different evaluators is shown in Table 2.

**Table 2:** The unprofessional behaviors of students from the viewpoints of different assessors.

Items		Clinical Teacher		Clinical Nurse		Student	
		No	Yes	No	Yes	No	Yes
1. Lack of maintaining medical dignity in their relationship, talking, dressing	N	108	32	102	38	105	35
	%	77.1	22.9	72.9	27.1	75.0	25.0
2. Denial of any errors, mistakes, and wrongdoing	N	85	55	98	42	106	34
	%	60.7	39.3	70.0	30.0	75.7	24.3
3. Dishonest behavior in the workplace	N	130	10	102	38	110	30
	%	92.9	7.1	72.9	27.1	78.6	21.4
4. Failure to comply with clinic regulations and policy	N	123	17	104	36	114	26
	%	87.9	12.1	74.3	25.7	81.4	18.6
5. Having personal conversations or making fun of students, other physicians, peers, or staffing the corridors of the clinic	N	101	39	36	104	77	63
	%	72.1	27.9	25.7	74.3	55.0	45.0
6. Eating or drinking in the hallway of the clinic	N	110	30	41	99	90	50
	%	78.6	21.4	29.3	70.7	64.3	35.7
7. Medical negligence in duties in the clinic setting	N	91	49	103	37	112	28
	%	65.0	35.0	73.6	26.4	80.0	20.0
8. Lack of observance of discipline in medical work	N	97	43	101	39	115	25
	%	69.3	30.7	72.1	27.9	82.1	17.9
9. Lack of commitment to be available and responsive when “on call.”	N	126	14	105	35	112	28
	%	90.0	10.0	75.0	25.0	80.0	20.0
10. Failure to perform duties in teamwork	N	77	63	107	33	119	21
	%	55.0	45.0	76.4	23.6	85.0	15.0
11. Failure to report the risky and/or inappropriate behavior of a colleague (after approaching the individual)	N	83	57	68	72	77	63
	%	59.3	40.7	48.6	51.4	55.0	45.0
12. Performing procedures without having sufficient skills (without supervision)	N	90	50	102	38	115	25
	%	64.3	35.7	72.9	27.1	82.1	17.9
13. Lack of commitment to continuous learning	N	76	64	102	38	113	27
	%	54.3	45.7	72.9	27.1	80.7	19.3
14. Disregard educational activities (e.g., arriving late to rounds for nonclinical reasons, skipping a lecture or seminars in which attendance is required)	N	128	12	103	37	119	21
	%	91.4	8.6	73.6	26.4	85.0	15.0
15. Lack of self-assessment and refusal to accept and apply constructive critiques	N	79	61	108	32	118	22
	%	56.4	43.6	77.1	22.9	84.3	15.7
16. Lack of equity and fairness in serving patients	N	107	33	114	26	121	19
	%	76.4	23.6	81.4	18.6	86.4	13.6
17. Lack of acceptance of probable health risks him/herself in front of the patient’s	N	95	45	104	36	117	23
	%	67.9	32.1	74.3	25.7	83.6	16.4
18. The lack of bearing, difficulty, and discomfort in responding to the medical needs of the patients	N	88	52	116	24	115	25
	%	62.9	37.1	82.9	17.1	82.1	17.9
19. Play down the feelings, needs, and wishes of the patient	N	107	33	111	29	123	17
	%	76.4	23.6	79.3	20.7	87.9	12.1
20. Lack of empathy and compassion with patients	N	107	33	113	27	124	16
	%	76.4	23.6	80.7	19.3	88.6	11.4
21. Prefer their interests to the interests of the patient	N	116	24	89	51	115	25
	%	82.9	17.1	63.6	36.4	82.1	17.9
22. Lack of commitment to patient privacy	N	131	9	123	17	125	15
	%	93.6	6.4	87.9	12.1	89.3	10.7
23. Lack of respect for people’s religious and cultural differences	N	117	23	48	92	69	71
	%	83.6	16.4	34.3	65.7	49.3	50.7
24. Addressing the patient inappropriately	N	128	12	119	21	127	13
	%	91.4	8.6	85.0	15.0	90.7	9.3
25. Lack of commitment to the privacy of the patient-physician relationship	N	125	15	123	17	130	10
	%	89.3	10.7	87.9	12.1	92.9	7.1
26. Not suggesting treatment options to patients who cannot afford them	N	114	26	120	20	125	15
	%	81.4	18.6	85.7	14.3	89.3	10.7
27. Failure to maintain a professional boundary in relation to patients or colleagues	N	137	3	120	20	129	11
	%	97.9	2.1	85.7	14.3	92.1	7.9
28. Failure to introduce yourself and the nurses and physician assistants to the patient and his family	N	55	85	82	58	118	22
	%	39.3	60.7	58.6	41.4	84.3	15.7

**Fig 1:**
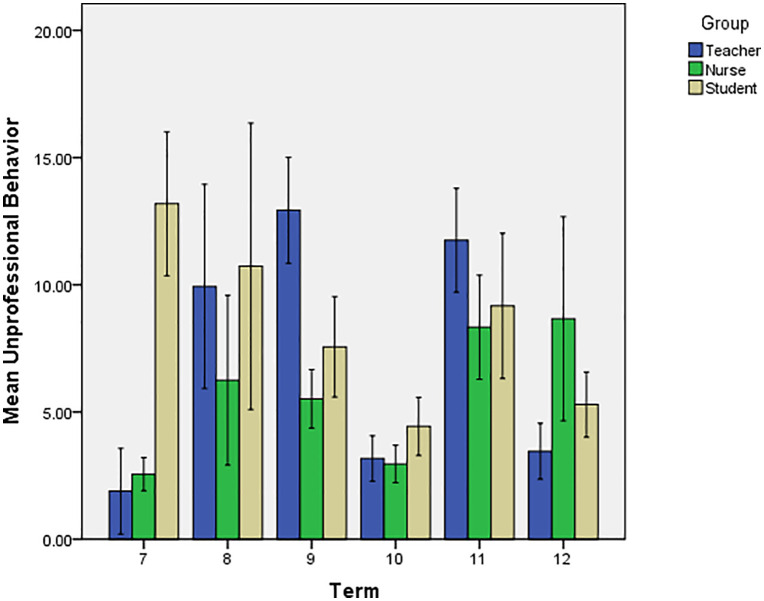
Participation in unprofessional behavior based on academic semesters.

The results showed that non-participation in unprofessional behaviors among students was higher according to the different evaluators’ responses. A significant difference has been reported between the total unprofessional behaviors from three groups of clinical teachers, nurses, and students’ self-assessments (P = 0.0001). The differences between clinical teachers’ and nurses’ opinions (P = 0.01) and nurses’ responses and students’ self-assessments (P = 0.0001) were significant. The unprofessional behaviors of the students in different semesters are displayed in Fig 1. The results indicated that the unprofessional behavior of students was significantly different between the three evaluator groups in the 7^th^ academic semester (P = 0.0001), the 9^th^ semester (P = 0.0001), and the 10^th^ semester (P = 0.018). The significant relationship between the age of participants and their unprofessional behavior was reported by the nurses’ responses (P = 0.01) and students’ responses (P = 0.002).

The results suggested that unprofessional behavior was reported more among men than women. This difference was significant from the nurses’ responses (P = 0.02) and students’ responses (P = 0.0001). (Table 3).

**Table 3:** The unprofessional behavior by gender in the viewpoints of different assessor groups.

Evaluator	Items	The engagement in unprofessional behavior		P-value
		Male	Female	
**Clinical Teacher**	Q3	7 (13.7)	3 (3.4)	0.02
	Q4	11 (21.6)	6 (6.7)	0.01
	Q16	17 (33.3)	16 (18)	0.04
**Nurse**	Q6	24 (46.2)	26 (29.5)	0.04
	Q12	16 (30.8)	9 (10.2)	0.002
	Q15	13 (25)	9 (10.2)	0.021
	Q16	11 (21.2)	8 (9.1)	0.045
	Q17	13 (25.0)	10 (11.4)	0.036
	Q18	17 (32.7)	8 (9.1)	0
	Q19	11 (21.2)	6 (6.8)	0.012
	Q20	12 (23.1)	4 (4.5)	0.001
**Students**	Q2	23 (42.6)	19 (22.1)	0.01
	Q4	20 (37)	16 (18.6)	0.01
	Q7	22 (40.7)	15 (17.4)	0.002
	Q13	20 (54)	18 (20.9)	0.03
	Q14	21 (38.9)	16 (18.6)	0.008
	Q17	23 (42.6)	13 (15.1)	0.00001
	Q20	15 (27.8)	12 (14)	0.04
	Q21	27 (50)	24 (27.9)	0.0008

### 3.2. Results of Quantitative phase

The current findings indicate that the students’ experiences regarding factors influencing unprofessional behavior among dental students can be categorized under the theme: “Disruption of Individual and Systemic Factors in the Development of Professional Behaviors.” This theme consists of two categories: “Individualism” and “Challenges of the Overt and Covert Curriculum in Professional Development” (see Table 4).

**Table 4:** Students’ experiences of factors affecting unprofessional behavior.

Subcategory	Category	Theme
Profiteering	**Individualism**	**Disturbance of individual and systemic factors in the development of professional behaviors**
Unwillingness to accept accountability for professional responsibility		
Driving effect of educational requirements and processes on engaging in unprofessional behaviors	**Challenges of the overt and covert curriculum in professional development**	
Negative role models of professional behavior		

#### 3.2.1. Individualism.

This category discusses personal factors affecting unprofessional behaviors, including the preference for individual interests, lack of recognition, and adherence to professional responsibilities.

**Profiteering**: The results explored that students’ preference for individual interests led to their engagement with unprofessional behaviors. Students stated they hide or do not express their mistakes or errors because they fear receiving negative feedback or reducing their score due to the mistake or error made. The students preferred that their profits, including personal prestige and score, were not jeopardized. Concerning the profiteering of learners in not expressing their errors, a student said:

*“My classmate did not accept his mistake. He feared ruining her character in front of his classmates and teacher, even if patient safety was also brought up*.” (Male student, 25 years old, 12^th^ semester).

**Unwillingness to accept accountability for professional responsibility**: In this subcategory, we discussed the neglect of professional responsibilities towards patients, the profession, and society. Boredom and a reluctance to face new challenges are key factors that lead students to be unwilling to accept patient care.

*“We visited numerous patients until we fulfilled all the educational requirements. We transported the additional patients who arrived after that. I don’t like to make trouble for myself, so that’s why.”* (Male student, 36 years old, 7^th^ semester).

The lack of understanding of responsibilities as a professional led to low students’ sensitivity to unprofessional behaviors. A participant stated:

*“I have nothing to do with the behavior of others. Everyone is treated according to their own opinion. The way people dress is based on their circumstances and family culture, which has nothing to do with me.”* (Male student, 25 years old, 9^th^ semester).

#### 3.2.2. Challenges of the overt and covert curriculum in professional development.

This category consists of two subcategories: “the influence of educational requirements and processes on unprofessional behaviors” and “negative professional role models.” The focus of this category is primarily on environmental and systemic factors.

**Driving effect of educational requirements and processes on engaging in unprofessional behaviors**: The participants believed that issues like time-limited educational programs, excessive educational requirements, and primarily quantitative assessments resulted from unprofessional behavior. Students often compromise professional principles to fulfill educational activities within a constrained timeframe or to meet their educational requirements. In this regard, a participant said:

*“The final examination is only taken by our teachers at our university. In my opinion, students gradually acquire the skills to achieve higher grades and demonstrate their capabilities as competent students. To get the best grade on the exam, they are instructed to do unethical things.”* (Male student, 25 years old, 13^th^ semester).

Students cannot fulfill their responsibilities due to limited working hours and limited access to equipment. A participant stated:

*“The nurses want students to leave when their work hours are over, even if the patient’s work is still left. Students are not treated well by the staff. They are unwilling to supply us and do not cooperate with us. They just treat your score as if it will be reduced.”* (Male student, 25 years old, 10^th^ semester).

**Negative role models of professional behavior**: The participants believed that the unprofessional behavior of clinical teachers is one of the influential factors in the prevalence of unprofessional behavior among them. A participant stated:

*“My clinical teachers are telling patients that they should endure pain and wait because they pay less here, which I hear. It is not permissible for you to protest. I convey the same message to my patient.”* (Male student, 25 years old, 12^th^ semester).

*“Many of my friends have mistreated patients’ teeth and did not follow the proper procedure. The student is afraid of getting scolded and disrespected by the teacher.”* (Female student, 28 years old, 14^th^ semester).

## 4. Discussion

The current findings indicate that unprofessional behavior was more prevalent in the areas related to responsibility and respect, as reported by all three groups: clinical teachers, nurses, and dental students. The highest frequency of unprofessional behavior was observed among junior learners and male students. Dental students described their experiences with factors influencing unprofessional behavior under the theme of “disruption due to individual and systemic factors affecting the development of professional behavior.” This was further categorized into “individualism” and “challenges posed by both the overt and covert curricula in professional development.”

The top three unprofessional behaviors of students reported by the clinical teachers’ responses include “failure to perform duties in teamwork, lack of commitment to continuous learning, and failure to introduce yourself and nurses and physician assistants to the patient and his family.” According to the nurses’ responses, the high frequency of the students’ unprofessional behaviors contains “having personal conversations or making fun of students, other physicians, and peers, staffing the corridors of the clinic, lack of respect for people’s religious and cultural differences, and eating or drinking in the hallway of the clinic”. The highest incidence of the unprofessional behaviors by students’ responses includes “having personal conversations or making fun of students, other physicians, and peers, staffing the corridors of the clinic, failure to report the risky and/or inappropriate behavior of a colleague, and lack of respect for people’s religious and cultural differences”. In sum, the highest frequency of unprofessional behavior was reported in the professional responsibility of the dental students in different situations.

Irresponsibility was introduced as one of the behaviors that can predict the unprofessional behavior of future healthcare providers [7]. The results indicated that the highest frequency of irresponsible behavior among dental students, as reported by evaluators, involved non-compliance with professional regulations—such as personal conversations and eating or drinking in the clinic—as well as non-disclosure of errors. These behaviors may stem from a lack of familiarity with professional duties and responsibilities among students.

The findings revealed that prioritizing personal interests and a reluctance to take responsibility for professional duties contribute to irresponsible behavior. The students reported engaging in unprofessional conduct to protect their individual interests. They cited factors such as fear of personal repercussions, negative reactions from clinical instructors, concerns about how others might judge them, and a competitive atmosphere as influences encouraging them to violate professional and ethical standards during treatment and education.

Participants expressed a willingness to hide or not disclose their mistakes due to fear of receiving negative feedback or affecting their grades. Essentially, dental students tend to prioritize their personal benefits when making decisions. Additionally, a lack of motivation to adhere to professional principles and limited awareness of unprofessional behavior in peers have contributed to the prevalence of such conduct within the dental school. An increased focus on personal gain, neglect of professional responsibilities, and an unsupportive organizational climate have raised concerns about the incomplete development of professional identity within educational systems. [27–29].

Furthermore, explicit challenges within the curriculum, such as cumbersome rules and disproportionately high educational requirements, lead students to compromise their professional principles when carrying out educational activities. The participants expressed that restrictions on working hours and limited access to necessary equipment forced them to violate key principles such as responsibility and altruism. As a result, they focused solely on fulfilling their educational requirements, often neglecting the quality of their work. This study explained the challenges posed by the educational system in developing professional behaviors, similar to findings presented in Mak-Van Der’s research. Unsupportive environments and educational bureaucracies were identified as context-based factors contributing to unprofessional behaviors [30]. Similar studies identified factors such as learners’ poor understanding of roles and responsibilities, along with a lack of comprehensive responses to patients and peers, as common causes of unprofessional behavior [7,31,32]. Chang et al. found that high student workload led to decreased adherence to professional responsibilities and reduced empathy towards patients in clinical setting^s^ [33]. In accordance with our findings, the category “the challenges of overt and covert curriculum” highlights an educational issue. In the study by Levinson et al., an unsupportive atmosphere was identified as a significant factor contributing to unprofessional behaviors [32]. The oversight of unprofessional behaviors in the educational system greatly affects their proliferation, undermines professional norms, and hinders the correction of such behaviors [18].

Disrespectful behaviors towards patients and colleagues were identified as significant examples of unprofessional conduct in the systematic review by Mak-van der Vossen [7]. In the present study, “disrespect for the patient’s culture and religion” had a high incidence based on the responses of nurses and students. Similarly, the most unprofessional behavior of medical students was reported in the “respect” domain by Jamal Abadi’s study [21]. The unprofessional behaviors in the respect domain may be caused by the adverse effects of the hidden curriculum and negative role models in the educational system in the investigated context. Respect components are mainly learned through students’ role models in the hidden curriculum process [34]. The negative impact of role models failing to adhere to professional principles, such as respect and responsibility, is examined as a systemic challenge. The hidden curriculum influences students’ learning through role models, organizational culture, and stereotyping [27]. Consistent with the current findings, Mak-van der Vossen identified negative role models as a significant factor in fostering unprofessional behaviors [7]. Our findings indicate that several factors contribute to the prevalence of unprofessional behavior in medical education systems. These include a lack of encouragement to uphold professionalism in the learning environment, the absence of rewards for professional conduct, and the non-punishment of unprofessional behaviors, as well as a general lack of commitment to professional principles among employees [31,32,35].

The findings indicated that unprofessional behaviors have a more significant impact on junior students. It was observed that the frequency of unprofessional behavior varied significantly across different academic semesters. According to responses from clinical teachers, the highest incidence of unprofessional behaviors was reported in the ^9th^ semester, while students reported a similar trend for the 7th semester. The educational curriculum of the investigated school requires students to complete numerous theoretical and practical units, along with research activities, including an undergraduate dissertation, during the 9th semester. This may contribute to an increase in unprofessional behavior among students during this time. In the qualitative phase of the study, several systemic challenges were identified as contributing factors to the prevalence of unprofessional behavior. These challenges include inadequate educational planning and the pressure to meet various requirements in both theoretical training and practical activities within the departments.

The high incidence of unprofessional behaviors observed among junior students in their 7^th^ semester may be attributed to the influence of negative role models, a lack of experience among students, and deficiencies in the curriculum regarding the teaching of professionalism principles. The participants believed that observing the disrespectful behavior of clinical teachers and seniors has normalized such behaviors among junior students and caused them to repeat unprofessional behaviors. Consistent with the present study, Jamal Abadi’s results showed that the unprofessional behavior rate was slightly higher among medical students than interns. However, this difference was not significant [21]. Similarly, Alfandre et al.’s study showed that engaging in unprofessional behavior did not increase among senior students [36]. These results may be attributed to negative learning experiences and the lack of professional development among junior students [37]. It is recommended to establish support mechanisms by discussing unprofessional behaviors, implementing monitoring, and providing feedback to enhance awareness of professional and unprofessional behaviors. Furthermore, developing compensation strategies for unprofessional behaviors within educational systems [7,30].

The results showed that the unprofessional behavior of male students was reported more than that of female students. Similarly, the findings of a study among West Virginia students showed that men had a lower tendency to label behaviors as unprofessional [38]. Elger showed that female clinicians could better recognize unprofessional behaviors, including behaviors related to the confidentiality of patient information and privacy [39]. The present results showed that unprofessional behaviors occur significantly more in men than in women in four items, including “failure to comply with clinical regulations and policy, lack of equity and fairness in serving patients, lack of empathy and compassion with patients, lack of acceptance of probable health risks by him/her in front of the patients.” The mentioned components were classified in the domains of altruism and responsibility. In line with our results, Pearson and colleagues showed a significant difference in the altruistic behaviors between men’s and women’s sensitivity to unprofessional behaviors [40]. Stratton et al. showed that men were less sensitive to respective behaviors than women [41]. Mak-van der Vossen suggested developing a mechanism of monitoring and constructive feedback to students, especially male students [30].

### 4.1. Strengths and limitations

The methodological approach used in this study, along with the identified framework of individual versus systemic factors, provides a valuable perspective for exploring these phenomena in other contexts. This could facilitate cross-cultural comparisons that help distinguish between universal challenges in professional identity formation and those that are specific to particular settings. However, it is important to note that this research was conducted within the unique socio-cultural and educational context of Iran’s dental education system. While core themes of unprofessional behavior—such as the tension between individualism and systemic challenges—are likely to be relevant globally, their specific manifestations and the emphasis on various factors (such as respect and responsibility) may be influenced by local cultural norms and institutional structures. Therefore, caution is recommended when attempting to generalize these findings to other cultural contexts without conducting further comparative studies.

This research was limited to one university, which affects the generalizability of the results and raises concerns about the external validity of the study. Additionally, observational assessments have inherent limitations that can impact results. To mitigate related biases, this study employed two trained evaluators in each department. Furthermore, the limitations of self-reporting can also affect the findings.

### 4.2. Implications of findings

The implications of these findings are diverse and emphasize the need for a focused effort to improve educational frameworks, offer targeted support, and develop an institutional culture that actively promotes professionalism as a fundamental competency. These results primarily encourage academic institutions to implement specific interventions for at-risk populations, incorporate explicit training in professional ethics, and transform the hidden curriculum through extensive faculty development and mentorship programs. This approach aims to systematically foster a culture of professionalism while addressing the identified contributing factors.

## 5. Conclusion

The most unprofessional behaviors reported among dental students were primarily related to issues of responsibility and respect. Qualitative findings suggested that both individual and systemic factors within dental schools contributed to the prevalence of these behaviors. On an individual level, a focus on profit and a reluctance to take responsibility for professional obligations hindered adherence to the principles of professionalism. At the systemic level, challenges arose from the hidden and learned curriculum in professional development, including complex and disproportionate educational requirements, training processes, and negative role models of professional behavior. It is recommended that both formal and informal programs be implemented to foster professional skills and increase awareness of unprofessional behaviors. Despite limited direct generalizability due to contextual differences, the individual-systemic disturbance model and hidden curriculum provide transferable insights, enabling other institutions to adopt MSF and early interventions targeting clinical role models and responsibility training.

## Supporting information

S1 Appendix Semi-structured interview guide. (DOCX)
