# Supplementary material for: Assessing unprofessional behaviors of dental students through multi-source feedback: A mixed methods analysis of engagement and influencing factors
Source: PLoS One. 2026 Jul 17;21(7):e0353159. doi: 10.1371/journal.pone.0353159 (PMC13379033; doi:10.1371/journal.pone.0353159)
Supplement: S1 Appendix — (DOCX) [file pone.0353159.s001.docx]

| **Supporting Information** |
| --- |
| **Appendix 1: Semi-Structured Interview Guide** |
| Study Title: Exploring Dental Students' Experiences with Unprofessional Behavior in Clinical Settings   \| **Interviewer____________**  **Date_____________**  **Time: Start: ________ \| End________**  **Participant Code_____________**  **Location_____________** \| \| \| --- \| --- \| \| **Pre-Interview** \| - Welcome participant and create comfortable atmosphere - Explanation of study purpose and confidentiality - Explain voluntary participation and right to withdraw at any time - Obtain written informed consent - Request permission to audio record the interview - Answer any questions before starting \| \| **Warm-Up Questions** \| - Tell me about your journey in dental school so far. (Year of study? Clinical rotations completed?) - How would you describe a "professional" dental student? What about "unprofessional behavior"? \| \| **Main Interview Questions** \| - Have you observed any unprofessional behaviors in the clinical setting? Please describe. - What types of unprofessional behaviors are most common among dental students? - What factors do you think cause dental students to behave unprofessionally? \| \| **Probing Questions** \| - Do stress, burnout, personal attitudes, or lack of knowledge play a role? - How do peer pressure, group norms, or competition influence behavior? - What about workload, time pressure, resources, or educational requirements? - How do clinical teachers and supervisors influence behavior? Any positive or negative examples? - How has the formal or informal curriculum prepared you for professionalism? Any gaps? - How would you describe the overall culture? Are there clear policies? What consequences exist? \| \| **General Probing Questions** \| - Can you tell me more about that? - What do you mean by that? - Can you give me a specific example? - How did others react? - How did that make you feel? \| \| **Section C: Responses & Improveme** \| - What happens when someone behaves unprofessionally? - If you could change one thing to promote professionalism, what would it be? - What advice would you give new students about professionalism? \| \| **Closing** \| - Summarize key points: "Let me briefly summarize what I heard from you today..." - Ask for confirmation: "Is this accurate? Have I missed anything important?" - Final question: "Is there anything else you would like to share about professionalism or unprofessional behavior that we haven't discussed?" - Express gratitude: "Thank you very much for your time and for sharing your valuable experiences." - Next steps: Explain how the data will be used and offer to share findings upon completion - Follow-up: Ask permission to contact again if clarification is \|  - Average interview time: 45 minutes |

| **Appendix 2 - The assessment of unprofessional behaviors** |
| --- |
| 1. Lack of maintaining medical dignity in their relationship, talking, dressing |
|  |
| 1. Denial of any errors, mistakes, and wrongdoing |
|  |
| 1. Dishonest behavior in the workplace |
|  |
| 1. Failure to comply with clinic regulations and policy |
|  |
| 1. Having personal conversations or making fun of students, other physicians, peers, or staffing the corridors of the clinic |
|  |
| 1. Eating or drinking in the hallway of the clinic |
|  |
| 1. Medical negligence in duties in the clinic setting |
|  |
| 1. Lack of observance of discipline in medical work |
|  |
| 1. Lack of commitment to be available and responsive when "on call." |
|  |
| 1. Failure to perform duties in teamwork |
|  |
| 1. Failure to report the risky and/or inappropriate behavior of a colleague (after approaching the individual) |
|  |
| 1. Performing procedures without having sufficient skills (without supervision) |
|  |
| 1. Lack of commitment to continuous learning |
|  |
| 1. Disregard educational activities (e.g., arriving late to rounds for nonclinical reasons, skipping a lecture or seminars in which attendance is required) |
|  |
| 1. Lack of self-assessment and refusal to accept and apply constructive critiques |
|  |
| 1. Lack of equity and fairness in serving patients |
|  |
| 1. Lack of acceptance of probable health risks him/herself in front of the patient’s |
|  |
| 1. The lack of bearing, difficulty and discomfort in responding to the medical needs of the patients |
|  |
| 1. Play down the feelings, needs, and wishes of the patient |
|  |
| 1. Lack of empathy and compassion with patients |
|  |
| 1. Prefer their interests to the interests of the patient |
|  |
| 1. Lack of commitment to patient privacy |
|  |
| 1. Lack of respect for people’s religious and cultural differences |
|  |
| 1. Addressing patient inappropriately |
|  |
| 1. Lack of commitment to the privacy of the patient-physician relationship |
|  |
| 1. Not suggesting treatment options to patients who cannot afford them |
|  |
| 1. Failure to maintain a professional boundary in relation to patients or colleagues |
|  |
| 1. Failure to introduce yourself and nurses and physician assistants to the patient and his family |
|  |
